# Immune Recovery Uveitis: Pathogenesis, Clinical Symptoms, and Treatment

**DOI:** 10.1155/2014/971417

**Published:** 2014-06-24

**Authors:** Beata Urban, Alina Bakunowicz-Łazarczyk, Marta Michalczuk

**Affiliations:** ^1^Department of Pediatric Ophthalmology and Strabismus, Medical University of Białystok, Białystok University Children's Hospital, Waszyngtona Street 17, 15-274 Białystok, Poland; ^2^Department of Pediatric Ophthalmology and Strabismus, Białystok University Children's Hospital, Waszyngtona Street 17, 15-274 Białystok, Poland

## Abstract

IRU is the most common form of immune reconstitution inflammatory syndrome in HIV-infected patients with cytomegalovirus retinitis who are receiving highly active antiretroviral therapy (HAART). Among patients with CMV in the HAART era, immune recovery may be associated with a greater number of inflammatory complications, including macular edema and epiretinal membrane formation. Given the range of ocular manifestations of HIV, routine ocular examinations and screening for visual loss are recommended in patients with CD4 counts <50 cells/*μ*L. With the increasing longevity of these patients due to the use of HAART, treatment of IRU may become an issue in the future. The aim of this paper is to review the current literature concerning immune recovery uveitis. The definition, epidemiology, pathophysiology, clinical findings, complications, diagnosis, and treatment are presented.

## 1. Introduction

The initial human immunodeficiency virus (HIV) infection is characterized by polyclonal activation of both T-lymphocytes and B-lymphocytes with the release of inflammatory cytokines. Patients exhibit an increase in the production of both CD4+ and CD8+ T-lymphocytes. T-lymphocyte turnover is promoted by the production of interleukin-6, interleukin-1, interleukin-2, and tumor necrosis factor (TNF)-*α*, all of which promote HIV replication [1]. This cascade further accelerates the destruction of the immune system. Advancing infection is accompanied by further CD4+ T-lymphocyte destruction and worsening of the immune status. As the HIV infection progresses, a loss of CD4+ memory cells accompanied by an inability to activate and afterwards replicate new CD4+ cells is observed. The progressive loss of CD4+ clones puts the patient at increasing risk of opportunistic infections [2, 3].

HIV infection and consequent activation of immune system cells remain the most common cause of ocular damage. The ocular microenvironment is both immunosuppressive and anti-inflammatory in nature. The retina is protected by the blood-retinal barrier (BRB) which is composed of human retinal microvascular endothelial cells (HRMECs) and retinal pigment epithelium (RPE). RPE acts as an important outer barrier to prevent the movement of pathogenic microorganisms (including HIV-1) from the blood into the eye. Ocular complications in HIV infection have been demonstrated to be closely related to the breakdown of the blood-retinal barrier (BRB); however, the underlying mechanism is not clear. Recently the role of Tat, the transactivator protein of HIV-1, which plays critical and complex roles in both the HIV-1 replication cycle and the pathogenesis of HIV-1 infection, is the object of research. HIV-1 Tat protein is released from HIV-infected cells and is found circulating in the blood of HIV-1-infected patients [4]. Findings of Chatterjee et al. indicated that the exposure of retinal neurosensory and glial cells to HIV Tat resulted in increased activation and release of proinflammatory mediators, predominantly the chemokine and neurotoxic factor CXCL10 and the cytokine TNF-*α* [5]. In addition to pro-inflammatory mediators, they observed that retinal cells also demonstrated increased cell activation as evidenced by augmented expression of GFAP, which is due to the activation of Muller glial cells. Recent study by Che et al. also emphasizes the role of HIV-1 Tat protein in the development of ocular complications during HIV infection [6]. HIV-1 Tat protein induced the apoptosis of human retinal microvascular endothelial cells and retinal pigment epithelium cells. In addition, they found that the activation of N-methyl-D-aspartate receptors (NMDARs) was involved in the apoptosis of RPE cells, but it caused no changes in HRMECs. Furthermore, both cell types exhibited enhanced expression of Bak, Bax, and Cytochrome c. The inhibition of Tat activity protected against the apoptosis induced by NMDAR activation and prevented the dysregulation of Bak, Bax, and Cytochrome c, revealing an important role for the mitochondrial pathway in HIV-1 Tat-induced apoptosis.

Immune reconstitution inflammatory syndrome (IRIS), previously known as immune restoration disease (IRD) or immune reconstitution syndrome (IRS), is characterized by paradoxical worsening of treated opportunistic infection or the unmasking of previously subclinical, untreated infection in patients with HIV after initiation of antiretroviral therapy [7–11]. Some individuals who are infected with HIV rapidly deteriorate shortly after starting antiretroviral therapy, despite effective viral suppression. This reaction, referred to as IRIS, is characterized by tissue-destructive inflammation. The current definition of IRIS includes five fundamental criteria: (1) confirmed case of HIV, (2) temporal association between development of IRIS and initiation of HAART (highly active antiretroviral therapy), (3) specific host responses to HAART, such as decrease in HIV viral load (plasma levels of HIV RNA) and an increase in CD4+ cell count, (4) clinical deterioration characterized by an inflammatory process, and (5) exclusion of other causes that may lead to a similar clinical presentation [7]. There are various manifestations of IRIS. Among the clinical features, frequently reported pathogens associated with IRIS are* Mycobacterium tuberculosis*, atypical mycobacterium, cytomegalovirus, varicella zoster virus, and* Cryptococcus neoformans*. However, there are some less common pathogens including* Pneumocystis jirovecii* pneumonia, toxoplasmosis, hepatitis B and C virus, MC and genital warts, sinusitis, and AIDS-related lymphoma [7, 10]. It has been proposed that IRIS is caused by a dysregulation of the expanding population of CD4+ T cells specific for a coinfecting opportunistic pathogen [10]. The interval between the initiation of HAART and the beginning of IRIS is highly variable (from 1 week to more than 1 year), but, in the majority of the cases, it occurs during the first two months of HAART [8]. Increased IL-8, Th1, and Th17 cytokine levels in IRIS patients precede ART initiation and could help identify patient populations at higher risk for IRIS [11]. Ocular IRIS is referred to as immune recovery uveitis (IRU). It remains a leading cause of ocular morbidity.

## 2. Etiology of IRU

With initial HIV infection T-lymphocyte activation and the production of proinflammatory lymphokines (e.g., Il-2, TNF-*α*, and IL-6) can be observed. T-lymphocytes, particularly the CD4+ fraction, are a primary target for HIV infection. They are destroyed as the HIV infection progresses [2]. As HIV disease continues and the number of CD4+ T-lymphocytes continues to decrease, the patient is at higher risk for the development of opportunistic infections, such as CMV retinitis. With the occurrence of acquired immunodeficiency syndrome (AIDS) number of cytomegalovirus retinitides (CMV retinitis) significantly increased [2, 12]. CMV retinitis is a complication of late-stage human immunodeficiency virus (HIV) infection and is associated with CD4+ T cell counts less than 50/*μ*L [13, 14]. CMV retinitis is the result of hematogenous spread of the virus to the retina through retinal blood vessels after systemic reactivation of a latent CMV infection. Patients with AIDS and CMV retinitis typically have minimal or no clinical vitritis or anterior chamber inflammation due to the underlying immunosuppressed state [15]. Even though anterior chamber is clinically quiet, a cytokine-mediated inflammatory response pattern is actively present in the aqueous humor. Cytokine analysis of aqueous humor in HIV patients with CMV retinitis revealed raised levels of IP-10, fractalkine, PDGF-AA, G-CSF, Flt-3L, and MCP-1 [16]. Higher median levels of these cytokines suggest a unique immunologic signature consistent with a combined Th-1 and monocyte macrophage-mediated response. The infection may initially be asymptomatic, but the retinal necrosis it produces may result in decreased visual acuity. A perivascular yellow-white retinal lesion frequently associated with retinal hemorrhage or a focal white granular infiltrate, often without hemorrhage, can be observed. Both lesions enlarge in a progressively expanding “brushfire” pattern [17].

In the era before highly active antiretroviral therapy, patients with CMV retinitis had minimal intraocular inflammation, and macular edema was rarely reported, but they required chronic suppressive anticytomegalovirus therapy to prevent relapse of the disease. If untreated, CMV retinitis spread throughout the entire retina, causing total retinal destruction and blindness. Highly active antiretroviral therapy (HAART) was introduced in 1996 to treat HIV-infected patients. It consists of combination of antiretroviral therapies. HAART includes one or two reverse transcriptase inhibitors and one or two protease inhibitors, recently expanded by an integrase inhibitor or an entry inhibitor (Table 1).

The most commonly used combination consists of one protease inhibitor and two reverse transcriptase inhibitors. This treatment leads to decreased plasma levels of HIV mRNA and increased CD4+ T-lymphocyte counts, resulting in increased patient survival and a decrease in the incidence of three major opportunistic infections:* Pneumocystis carinii* pneumonia,* Mycobacterium avium* complex disease, and cytomegalovirus retinitis [18, 19]. The first phase of immune recovery after initiation of HAART is characterized by a redistribution of both naïve and memory CD4+ T cells from lymphatic tissues, whereas the subsequent gradual CD4+ T cell recovery over time is primarily naïve CD4+ cells [20]. With HAART therapy the incidence of CMV retinitis has decreased by 80% to 90%, but it has not dropped to zero [21]. Before the availability of HAART, a diagnosis of CMV retinitis required anti-CMV therapy, which was associated with severe morbidity and was very expensive; the annual cost of oral ganciclovir for one patient was 17,000 $ in 1998 [18]. A response to HAART is defined as an increase in CD4+ T cell count of at least 50 cells/*μ*L to a level of 100 cells/*μ*L or more. Unfortunately, HAART fails in up to 50% of AIDS patients due to noncompliance, side effects of the drugs, adverse drug interactions, or HIV resistance.

## 3. Definition of IRU

In 1998 Karavellas et al. and Zegans et al. have described a new intraocular inflammatory syndrome, which develops in patients with AIDS and inactive CMV retinitis, who have experienced HAART-mediated increases in CD4+ T-lymphocyte levels [15, 22]. This syndrome was initially called immune recovery vitritis because the report by Zegans et al. describes transient vitritis as the principal manifestation. Karavellas et al. defined IRU as vitritis of 1+ or greater severity with visually important floaters, a decrease in vision of 1 or more lines, or both, with or without associated papillitis and macular changes [12].

Currently, there have been no definite criteria of IRU [23]. It is generally recognized by new or increased noninfectious intraocular inflammatory reaction in patients with AIDS and cytomegalovirus retinitis several weeks after starting HAART. The inflammation is associated with an increase in the CD4+ T-lymphocyte counts of at least 50 cells/mm^3^ to the level of 100 cells/mm^3^. Time from beginning of HAART till an increase in CD4+ count is approximately 2 months [22]. IRU is predominantly a posterior segment inflammatory disorder that results in decreased vision and floaters in the affected eye [22]. It is currently one of the most common causes of new vision loss in patients with AIDS-related CMV retinitis [24].

The current definition of IRU (similarly IRIS) includes at least five main criteria: (1) being a patient with AIDS, (2) receiving HAART, (3) achieving an immune reconstitution indicated by increased CD4+ T cell count over 100 cells/mm^3^ for at least two months, (4) having preexisting CMV retinitis which is currently in the inactive state, and (5) developing an intraocular inflammation that cannot be explained by drug toxicity or a new opportunistic infection.

The severity of inflammation is related to various factors such as extent of CMV retinitis, amount of intraocular CMV antigen, degree of immune constitution, and previous treatment [19]. IRU usually develops in patients with inactive CMV retinitis but it rarely can occur in eyes with active CMV retinitis, especially at the onset of inflammation [25]. IRU usually affects all eyes with CMV retinitis, but sometimes patients had IRU in only one of the two eyes with CMV retinitis (26.3% of those with bilateral retinitis in the study of Kempen et al.) [26].

## 4. Pathogenesis of IRU

Although the pathogenesis of IRU is not entirely certain, it appears to represent an inflammatory reaction to either cytomegalovirus antigen in the eye or low or subclinical levels of cytomegalovirus replication and this inflammatory reaction occurs as the immune system recovers competency [13, 15, 27].

Since IRU in non-CMV retinitis eyes is not common, the ocular inflammation is postulated to be due to the CMV infection itself, which causes breakdown in the blood ocular barrier. This may allow CMV antigens to leak out of the eye and give the antigen access to lymphoid organs and stimulate an antigen-specific immune response [19, 28].

Immunohistological examination of epiretinal membrane associated with IRU showed evidence of chronic inflammation with predominant T-lymphocytes. This data, in conjunction with the finding of a positive correlation between IRU and surface area of inactive CMV retinitis, would suggest that IRU may be due to T cell-mediated reaction to CMV antigen present in inactive CMV retinitis [29].

According to Nussenblatt and Lane, as immune function after HAART improves, a threshold is reached at which the body can mount an intraocular inflammatory response to cytomegalovirus antigens present in the eye [2]. With continued recovery of immune function, a higher threshold is reached at which the immune system inactivates cytomegalovirus, production of antigen stops, and inflammatory reactions subside.

Speculation on the pathophysiology of IRU includes the fact that the intraocular inflammation is a reaction to antigenically altered retinal or glial cells adjacent to the healed CMV lesion or secondary to chronic subclinical viral replication along the border of healed CMV [30].

Although the pathologic immune reaction in IRU occurs in the eye, some kind of immune dysregulation that allows for the development of pathologic response is likely caused by faulty systemic immune cell reconstitution [31]. IRU, just like IRS, could be a result of unbalanced reconstitution of effector and regulatory T cells, leading to exuberant inflammatory response in patients receiving HART. Biomarkers, including interferon-*γ* (INF-*γ*); tumour necrosis factor-*α* (TNF-*α*); C-reactive protein (CRP); and interleukin- (IL-) 2, -6, and -7, are subject of intense investigation at present [32].

Schrier et al. examined aqueous and vitreous fluids from patients with IRU and active CMV retinitis for the presence of cytokines, using enzyme-linked immunosorbent assay techniques, and CMV DNA by polymerase chain reaction [28]. They observed that IRU eyes had the highest levels of IL-12 (median 48 pg/mL), moderate levels of IL-6 (median 146 pg/mL), and low interferon gamma (median 15 pg/mL) compared to control. Additionally all uveitis eyes were CMV DNA negative; in contrast eyes with active CMV retinitis were CMV DNA positive. They concluded that inflammatory IRU can be differentiated from active CMV retinitis by the presence of IL-12 and less IL-6 and absence of detectable CMR replication.

Hartigan-O'Connor et al. in a multicenter observational study studied T_reg_ cell control over T cell responses in peripheral blood mononuclear cells from 25 patients with CMV retinitis and IRU and 49 immunorestored by HAART control subjects with CMV retinitis who did not develop IRU [31]. They observed weak antiviral CD4+ T cell responses in patients with IRU, as compared with control subjects, whereas CD8+ T cell responses were comparable. They also found that patients with IRU were characterized by a smaller number of Th17 cells (identified by measuring IL-17 production) than control subjects. They speculate that lower numbers of Th17 cells among patients with IRU may reflect greater losses throughout the course of HIV disease and a greater level of immune dysfunction. In their opinion CD4 cell count and Th17 cell number may both be measures of the severity of HIV disease before the initiation of HAART.

Schrier et al. after examining aqueous and vitreous fluids from patients with IRU and active CMV retinitis observed that IRU can be differentiated from active CMV retinitis by the presence of IL-12 and less IL-6 and absence of detectable CMV replication [28]. Increased levels of proinflammatory cytokines have also been documented in tissues of patients who have recovered from CMV retinitis. The upsurge of macular and disc edema seems associated with the production of interleukin-4 and tumor necrosis factor alpha, whereas vitritis is associated with the production of interleukin-2 and interferon gamma [33].

A report of Modorati et al. suggested that all patients presenting with the clinical and ophthalmological characteristics of IRU showed the presence of HLA B 8–18 [34].

## 5. Occurrence of IRU 

The median time from HAART initiation to develop IRU has varied from 20 to 43 weeks [4]. The median time to develop IRU in a study by Karavellas et al. was 45 weeks [27]. Hartigan-O'Connor et al. observed that median interval between diagnosis of CMV retinitis and IRU diagnosis was 47.5 months (range from 3 to 128 months) [31]. The study by Sudharshan et al. noted that interval between the start of HAART and onset of IRU was from 4 months to 2.5 years [35].

Kempen et al. evaluated the prevalence of immune recovery uveitis (IRU) in eyes of 374 patients with AIDS and CMV retinitis [26]. 36 patients (9.6%) were diagnosed with IRU in this 19-clinical-center cohort study. In the study by Karavellas et al. the prevalence of IRU varied from 38% to 63% in patients with CMV retinitis [27]. In India CMV retinitis still remains the commonest ocular manifestation in AIDS cases. In the study by Sudharshan et al. who examined 1000 HIV patients, the incidence of CMV retinitis remains high (36.2%) even in the era of HAART [35].

The occurrence of IRU appears to vary between studies, and the reason for this variability is unclear [13, 26, 27, 35–45]. Prevalence of IRU is presented in Table 2.

The degree of immune recovery may explain some of this variability [19]. Immediately after the introduction of HAART, the incidence of IRU based on large single-center cohort studies differs substantially, ranging from 0.11 per person-year (PY) to 0.83/PY [13, 27, 37]. One of the reasons of this disparity can be the role of intravitreal cidofovir, which is a major risk factor for IRU, and it was used in the treatment for CMV retinitis in the older studies [27]. The next reason is the time of starting HAART therapy in a patient with active CMV retinitis. Ortega-Larrocea et al. noted that early introduction of HAART in patient with CMV retinitis before completing induction therapy for CMV results in a higher incidence of IRU (71%) than among those who had suppressed CMV retinitis before starting HAART (31%) [38]. This data suggests that all patients with CMV retinitis should be treated for CMV and HAART therapy should be delayed until treatment of CMV retinitis is completed. The lower incidence of IRU in some studies might be related to more aggressive anticytomegalovirus therapy before and immediately after initiation of potent antiretroviral therapy, thereby minimizing exposure to CMV antigens during a critical phase of immune recovery in the eye [30]. Jabs et al. tried to describe in the prospective, multicenter observational study the five-year outcomes of patients with CMV retinitis and AIDS in the era of HAART [21]. They observed that the rate of IRU was 1.7/100 PY and varied from 1.3/100 PY for those with previously diagnosed retinitis and immune recovery at enrollment to 3.6/100 PY for those with newly diagnosed retinitis who subsequently experienced immune recovery. Despite the availability of HAART, patients with AIDS and CMV retinitis are at increased risk for mortality, retinitis progression, complications of the retinitis, and visual loss over a 5-year period.

The other explanation for variability of IRU occurrence may be some genetic or environmental differences, which might influence the susceptibility to CMV retinitis and then IRU. It is not yet possible to identify at-risk patients on the basis of laboratory tests of immune function.

## 6. Signs and Symptoms of IRU

Immune recovery uveitis may dramatically change the clinical situation in some patients. Many investigators observed that, instead of CMV necrotizing retinitis, inflammatory involvement can lead to intraocular disorders. The clinical picture of IRU is still evolving. The severity of the inflammation depends on the degree of immune reconstitution, extent of CMV retinitis, amount of intraocular CMV antigen, and previous treatment.

Symptoms typically include floaters and/or vision loss, the latter usually of moderate degree, with visual acuities worse than 20/40 but better than 20/200 [12, 14, 19, 26, 46].

IRU manifests symptomatically with decreased vision and/or floaters. Within weeks after starting HAART and rising CD4+ T-lymphocyte count, an exudate in the anterior chamber and vitreous haze appears. Because of its transient nature, this stage can be missed by the clinician. The inflammatory reactions may improve, but in some patients the uveitis will develop and may be complicated by, for example, papillitis and macular changes [3, 13].

### 6.1. Mild to Severe Vitritis

Canzano et al. observed that, after resolution of vitritis, vitreomacular traction syndrome (VMT) may be developed [47]. They speculated that changes in immune status may permit an inflammatory response that can lead to VMT. Henderson and Mitchell reviewed charts of 80 patients with inactive CMV retinitis, who received HAART treatment [14]. In most of these patients a mild transient vitritis was observed, which did not require treatment. IRV developed in only 7 patients significantly enough (based on deteriorating visual acuity) to require therapy. The nine eyes involved with significant IRV had a mean visual acuity loss of 2.8 Snellen lines.

### 6.2. Cystoid Macular Edema (CME)

CME is a complication that can result from this inflammation and is emerging as a major cause of visual loss in human immunodeficiency virus- (HIV-) infected patients. The main symptoms are decreased vision, metamorphopsia, and floaters [48, 49]. In the study by Kempen et al. eyes with IRU had a 20-fold higher risk of CME [26].

### 6.3. Epiretinal Membrane Formation

Kempen et al. observed that eyes with IRU had a 5- to 6-fold higher risk of epiretinal membrane than eyes without IRU [26]. Immunohistological examination of epiretinal membrane associated with IRU showed evidence of chronic inflammation with predominant T-lymphocytes [46].

### 6.4. Frosted Branch Angiitis

Frosted branch angiitis is essentially a severe form of vasculitis which affects the entire retina. It is commonly associated with cytomegalovirus infection and the administration of anticytomegalovirus therapy without the need for corticosteroids [50]. In patients with IRU, frosted branch angiitis can occur in the eye with active CMV retinitis and can be unilateral or bilateral. Leeamornsiri et al. described a 40-year-old woman with AIDS and CMV retinitis, who was treated with intravitreal injection of 2 mg/0.04 mL ganciclovir, and retinitis had improved [23]. One week after HAART initiation, while cytomegalovirus was not completely resolved, extensive frosted branch angiitis was noted. 25 mg/day oral prednisolone was given with continuation of HAART and intravitreal ganciclovir and such treatment leads to significant improvement of perivascular infiltration within one week and this case has reported the earliest onset of IRU after HAART initiation. Recently Alp et al. described frosted branch angiitis associated with HAART in patient with immune recovery uveitis despite a low CD4+ T cell count (20 cells/mm^3^) [51].

### 6.5. Papillitis

See [12, 17, 27].

### 6.6. Neovascularization of the Retina or Optic Disc [13, 52, 53]

Wright et al. reported extensive peripheral retinal neovascularization as a late finding of IRU in 3 HIV-infected patients with inactive CMV retinitis, 1 of whom developed recurrent vitreous hemorrhage that required vitrectomy [53]. The pathogenesis of fibrovascular membrane formation in patients with IRU may be hard to elucidate, because it is relatively uncommon and because surgically obtaining tissue specimens is not justified if the membrane follows a benign clinical course. Specific therapy for these fibrovascular membranes is not required unless they lead to recurrent vitreous hemorrhages and vision loss.

### 6.7. Proliferative Vitreoretinopathy with Retinal Detachment

In the study by Karavellas et al. 29 eyes of 21 patients with IRU and inactive CMV retinitis were followed up for median of 43 weeks after diagnosis of IRU [12]. Four eyes developed clinically important posterior segment complications. Two of these eyes had extensive proliferative vitreoretinopathy which developed within 2 to 3 days after rhegmatogenous retinal detachment. Immunostaining of proliferative vitreoretinopathy membranes from eyes with IRU revealed numerous lymphocytes, the majority of which were positive for T-lymphocytes cell markers, indicating that epiretinal proliferation in these eyes is the result of an inflammatory process in which T-lymphocytes play a role. All the eyes with this complication had a poor final visual outcome. One eye developed a vitreous hemorrhage from avulsion of a blood vessel secondary to contraction of the inflamed vitreous and partial posterior vitreous detachment. Additionally one eye developed extensive epiretinal and subretinal proliferation.

### 6.8. Anterior Segment Inflammation, Iris Synechiae, and Cataract

From the experience of Holland, patients with IRU-associated cataracts are particularly prone to postoperative problems such as posterior synechiae, papillary membranes, and inflammatory deposits on the lens implants [3]. In the study by Karavellas et al. anterior segment complications developed in seven eyes of twenty-nine eyes with IRU. These complications included progressive posterior subcapsular cataracts, anterior subcapsular cataract, and persistent postoperative anterior chamber inflammation with development of posterior synechiae and large visually important inflammatory deposits on the surface of the intraocular lens [12]. The authors assumed that subcapsular opacification of the lens in some eyes with IRU is a multifactorial process, involving such factors as corticosteroid therapy and previous surgery. It is also possible that patients with IRU may develop more severe and/or prolonged inflammation after intraocular surgery.

### 6.9. Panuveitis with Hypopyon

See [25, 54].

### 6.10. Macular Hole

See [37].

### 6.11. Cytomegalovirus Immune Recovery Retinitis (CMV-IRR)

Recently, Ruiz-Cruz et al. reviewed charts of 75 patients with CMV retinitis on HAART initiation or during the 6 subsequent months [55]. 20 patients had improvement of CMV retinitis. The remaining 55 patients experienced CMV-IRR; 35 of those developed CMV-IRR after HAART initiation (unmasking CMV-IRR); and 20 experienced paradoxical clinical worsening of retinitis (paradoxical CMV-IRR). Nineteen patients with CMV-IRR had ≥50 CD4 T cells/mm^3^. Six patients with CMV-IRR subsequently developed immune recovery uveitis (IRU). The authors propose definition for CMV-IRR as the condition which is likely to occur after successful initiation of HAART, even in patients with high CD4 T cell counts.

## 7. Risk Factors

The first well-known risk factor for IRU is immune recovery with a rapid rising in the number of CD4+ T-lymphocytes as a consequence of HAART [23]. The risk of IRU increases manyfold with increasing CD4+ T cell count to a level of ≥100 cells per microliter or decreasing HIV load [3, 22, 24, 26, 32].

Song et al. observed that the use of intravenous cidofovir is a primary risk factor in the subsequent development of immune recovery uveitis [56]. They assume that ongoing treatment of healed CMV retinitis after immune recovery does not appear to protect against the development of immune recovery uveitis. Kempen et al. reported that the use of intravitreous injections of cidofovir was associated with a 19-fold higher risk of IRU [26]. Similar observations were made by Kempen et al. [26].

Another risk factor for IRU includes surface area of retinal involvement due to CMV retinitis [26]. Karavellas et al. suggested that a higher antigen load in larger lesions would increase the likelihood that IRU would become clinically manifest [46]. In their study patients with >30% of retinal area affected had 4.5-fold higher risk of developing IRU when compared with eyes with a retinal CMV area of <18%. Effects of lesion size on the extent of blood-retinal barrier breakdown are also a potential explanation of this association. In contrast, Arevalo et al. observed that eyes with IRU had a mean CMV surface area of 31.7% and eyes without IRU (control group) had a mean CMV surface area of 35%, so in their opinion CMV surface area does not seem to be a risk factor for the development of IRU [37]. Initiation of HAART should be delayed until after the induction phase of anti-CMV therapy, as the reduction of antigen load with anti-CMV agents may reduce potential risk of IRU [3].

On the other hand, the presence of a posterior pole lesion and male gender were found to be associated with reduced IRU risk [26].

Some other unidentified factors may influence susceptibility and severity of IRU, so further research to identify such factors is desired.

## 8. Treatment of IRU

The treatment of IRU depends on the location of the intraocular inflammation, the severity of the inflammation, and the presence of ocular complications, particularly CME.

### 8.1. Pharmacological Treatment

Inflammation in the anterior chamber is treated with topical corticosteroids in frequencies typical of treating other forms of anterior uveitis [14]. If IRU is an isolated mild vitritis without CME, these eyes may be observed, as the vitreous inflammation can be transient. Immune recovery uveitis with more severe vitreous inflammation and/or CME typically is treated with periocular corticosteroids (triamcinolone acetonide 40 mg), or short courses of oral corticosteroids, without recurrence of the CMV retinitis [46]. The main advantage of periocular corticosteroids is the production of therapeutic local drug levels to avoid the potential problems of systemic corticosteroids in these immunosuppressed patients [3, 21, 24].

Intravitreal corticosteroids have successfully treated eyes with IRU, refractory to less aggressive treatment; however, in addition to the usual complications of cataracts and glaucoma, reactivation of retinitis may occur [57]. To prevent CMV reactivation following corticosteroid treatment, some authors recommend restarting anti-CMV therapy [3]. Anti-CMV therapy is important during immune recovery because it has been proved to be protective against the development of IRU by reducing the amount of CMV antigens in the retina, although it has not shown a favorable cost-effectiveness ratio where there are no signs of CMV retinitis [3]. Kuppermann and Holland suggested that continued, aggressive anticytomegalovirus therapy for a prolonged time after initiation of potent antiretroviral therapy may reduce the rate or severity of IRU [30]. There are many anti-CMV drugs, which are available in intravenous, oral, and intravitreous therapy [13, 15, 19, 25, 26, 51]. Ganciclovir is the first anti-MCV drug, available since 1984. To achieve high tissue concentrations during induction, ganciclovir is administered intravenously (Cytovene) twice daily at a dose of 5 mg/kg. Foscarnet (Foscavir) is generally considered a second line intravenous therapy that is often administered to patients with ganciclovir-resistant viral strains or dose-limiting neutropenia. Cidofovir (Vistide), the third intravenous drug, because of its association with immune recovery uveitis, should not be used if immune recovery is expected. Oral ganciclovir was introduced in 1994 in attempt to lower costs, to eliminate the inconvenience of daily intravenous drug injections, and to improve patient quality of life. The primary indication for oral ganciclovir was prevention of contralateral retinitis and nonocular CMV disease in patients receiving intraocular therapy [30]. When oral valganciclovir (Valcyte) with its high bioavailability and convenient once-daily dosing was introduced, production of oral ganciclovir was discontinued. Intravitreal ganciclovir injections are given to patients who are intolerant of or refused systemic therapy. After cidofovir intravitreal injections uveitis occurred frequently, so this kind of administration is no longer recommended. The most popular in the industrialized world is intraocular implant of ganciclovir (Vitrasert), which produces intraocular levels of ganciclovir five times that of systemically administered ganciclovir and allows for avoidance of systemic toxicity [58].

Figueiredo et al. described IRU masked as endogenous endophthalmitis and hypopyon, treated with oral valganciclovir and topical dexamethasone [25]. The authors assume that maintenance treatment should be continued until immune recovery is achieved, because none of the anti-CMV drugs available eradicate ocular and systemic CMV antigens in the immunocompromised patient.

Vision loss in patients with IRU is usually caused by macular pathology, primarily cystoid macular edema. Several treatment options for patients with IRU and macular edema have been proposed. Physicians previously suggested the use of oral corticosteroids [17, 24]. However, Karavellas et al. reported the use of repository sub-Tenon steroid injections for the treatment of macular complications in IRU and they found only a modest effect [46]. Similarly, in the study by Nguyen et al. 4 eyes were reported to have IRU associated with CME, and CME improved in 2 of them (50%) [13]. In the other two patients, the CME persisted despite aggressive therapy with topical, periocular, and systemic corticosteroids. These results are also in agreement with other clinical studies [14, 37]. In the study of Kosobucki et al. 5 patients with chronic macular edema as a result of IRU were examined [29]. Fluorescein angiography, visual acuity, and CMV lymphoproliferative T cell function assays were obtained after receiving valganciclovir 900 mg daily for three months and again three months after withdrawal of therapy. Their vision improved by a mean of 11 letters, angiograms showed reduction of macular edema, and hematologic and CD4 count data remained stable. The authors assume that the lack of significant decrease in CMV lymphoproliferative response suggests that if valganciclovir is suppressing residual CMV replication, it is not reducing the cellular immune response to CMV. Morrison et al. used intravitreal injection of 20 mg decanted triamcinolone acetate (IVTA) for the treatment of macular edema secondary to IRU and visual acuity improved in all patients [59]. In total, 8 eyes of 7 patients received 13 injections. Visual acuity and OCT retinal volume and thickness improved in all patients, but longer follow-up is needed to assess the durability of the effect and to monitor for longer-term complications (the risk of the formation of cataract, glaucoma, and endophthalmitis). The authors observed no cases of cytomegalovirus reactivation during a minimum follow-up of 9 months. This kind of therapy allows for avoidance of the side effects of systemic oral corticosteroid treatment. Mild inflammation with macular edema can sometimes be treated effectively with topical and periocular corticosteroids, but other eyes are refractory to treatment [14, 46, 57]. El-Bradey et al. after examining long-term results of treatment of macular complications in eyes with IRU noted that mild cases of immune recovery uveitis and macular edema may be observed [57]. They observed that, in the eyes with reduction of vision due to cystoids macular edema, there was only a modest treatment effect using repository corticosteroids. These patients with more severe inflammatory changes with VA of 20/30 or worse due predominantly to CME were treated with a series of posterior sub-Tenon injections of repository corticosteroids. In this study, repository corticosteroids appeared to improve vitritis with decline in inflammatory cells in 60% of treated eyes, but this treatment had a lesser effect on the visual acuity, which improved in only 40% of the treated eyes. In addition, macular edema was resistant to corticosteroid injections. Henderson and Mitchell reported successful treatment of nine eyes of seven patients of immune recovery vitritis with orbital floor injections of methylprednisolone acetate 40 mg or triamcinolone 20 mg [14]. Four of these nine eyes had CME, which showed improvement or disappearance after this treatment, and no complications were reported.

Recent studies have demonstrated that intravitreal immunosuppressant injections of methotrexate or anti-VEGF agents may not only lead to fewer intraocular side effects but also have a lower therapeutic activity for the reduction of macular edema in uveitis, because blockage of VEGF has not been shown to have an anti-inflammatory effect [60].

Recently, fluocinolone acetonide (Retisert) was used to treat cystoid macular edema resulting from IRU [61]. Improvement was observed in two of three eyes and no CMV reactivation was detected during the several-month follow-up period.

The most recent study by Krishnan and Chatterjee showed that endocannabinoids (N-arachidonoylethanolamide and 2-arachidonoylglycerol) could be used to alleviate Tat-induced cytotoxicity during HIV infection and rescue retinal cells [62]. The neuroprotective mechanism involved suppression in production of proinflammatory and increase in anti-inflammatory cytokines, mainly through the MAPK pathway. Both endocannabinoids regulated cytokine production by affecting at the transcriptional level the NF-*κ*B complex, including IRAK1BP1 and TAB2. These findings have direct relevance in immune recovery uveitis where antiretroviral therapy has helped immune reconstitution. In their opinion endocannabinoids and their agonists may be thought of as neurotherapeutic during certain conditions of HIV-1-induced inflammation. Also recent findings of Che et al. related to the role of HIV-1 Tat protein in breaking the blood-retinal barrier suggest that the inhibition of HIV-1 Tat activity could be essential in the future therapy of CME secondary to IRU [6].

### 8.2. Surgical Treatment of IRU

Treatment with corticosteroids (subtenon or systemic or intravitreal) is effective in controlling inflammation and improving vision in some cases. However, surgery may be required in patients with vitreomacular traction syndrome, epiretinal membrane formation, cataract, and proliferative vitreoretinopathy. El-Bradey et al. observed that, in eyes with structural macular changes secondary to IRU, such as dense epiretinal membrane (ERM), trans- pars plana vitrectomy with peeling of ERM resulted in vision improvement in three of four eyes, but the cystoid macular edema persisted despite surgery [57]. The effect of vitrectomy on inflammatory cystoid macular edema is not yet clear and might become more important in the future [63].

## 9. Summary

Among patients with CMV in the HAART era, immune recovery may be associated with a greater number of inflammatory complications, including macular edema and epiretinal membrane formation. Given the range of ocular manifestations of HIV, routine ocular examinations and screening for visual loss are recommended in patients with CD4 counts <50 cells/*μ*L. As studies on HIV disease after the introduction of HAART continue to become available, more thorough descriptions of treated patients with ocular opportunistic infections will include side effects and toxicities on therapy. As increasing number of HIV-infected individuals present with treatment failure in developing countries, the risk of ophthalmic complications may increase. With the increasing longevity of these patients due to the use of HAART, treatment of IRU may become an issue in the future.

## Figures and Tables

**Table 1:** Kinds of antiretroviral drugs used in HAART therapy.

**Reverse transcriptase inhibitors (RTIs)**	
(1) Nucleoside and nucleotide analog reverse transcriptase inhibitors (NRTIs)	
Abacavir (Ziagen)	
Abacavir + lamivudine + zidovudine (Trizivir)	
Abacavir + lamivudine (Epzicom/Kivexa)	
Didanosine (Videx, Videx EC)	
Emtricitabine (Emtriva)	
Lamivudine (Epivir)	
Lamivudine + zidovudine (Combivir)	
Stavudine (Zerit)	
Tenofovir (Viread)	
Zalcitabine (Hivid)	
Zidovudine (Retrovir)	
(2) Nonnucleoside reverse transcriptase inhibitors (NNRTIs)	
Delavirdine (Rescriptor)	
Efavirenz (Sustiva, Stocrin, Efavir)	
Efavirenz + tenofovir + emtricitabine (Atripla)	
Etravirine (Intelence)	
Nevirapine (Viramune)	
Rilpivirine (Edurant)	
Rilpivirine + tenofovir + emtricitabine (Complera)	

**Protease inhibitors (PIs)**	
Amprenavir (Agenerase)	
Atazanavir (Reyataz)	
Darunavir (Prezista)	
Fosamprenavir (Lexiva, Telzir)	
Indinavir (Crixivan)	
Lopinavir (Kaletra)	
Nelfinavir (Viracept)	
Ritonavir (Norvir)	
Saquinavir (Fortovase, Invirase)	
Tipranavir (Aptivus)	

**Integrase inhibitors**	
Raltegravir (Isentress)	

**Entry inhibitors**	
Maraviroc (Selzentry)	
Enfuvirtide (Fuzeon)	

**Table 2:** Occurrence of IRU in patients starting HAART for HIV infection.

Authors of the study	Country	Year of publication	Patients with IRU
Karavellas et al. [27]	USA	1999	63.3%
Nguyen et al. [13]	USA	2000	18.2%
Banker and Patel [36]	India	2002	41.7%
Arevalo et al. [37]	Wenezuela	2003	37.5%
Ortega-Larrocea et al. [38]	Mexico	2005	53.5%
Kempen et al. [26]	USA	2006	9.6%
Uemura et al. [39]	Japan	2006	30%
Dujić and Jevtović [40]	Serbia	2007	42.9%
Lin et al. [41]	Taiwan	2008	24.4%
Gharai et al. [42]	India	2008	5%
Shah et al. [43]	India	2009	3%
Hamamotoo et al. [44]	Japan	2012	1.5%
Sudharshan et al. [35]	India	2013	17.4%
Agarwal et al. [45]	India	2014	33.33%
